# Systemic p-ANCA vasculitis with fatal outcome, arising in the setting of methimazole use 

**DOI:** 10.5414/CNCS109759

**Published:** 2019-04-16

**Authors:** Sean Hacking, Nupur N. Uppal, Neelofar Khan, Marina Ionescu, Vanesa Bijol

**Affiliations:** 1 Department of Pathology and Laboratory Medicine and; 2 Division of Nephrology and Hypertension, Zucker School of Medicine at Hofstra Northwell, Manhasset, NY, USA

**Keywords:** methimazole, ANCA, vasculitis, drug-induced vasculitis

## Abstract

Here we report a fatal case of antineutrophil cytoplasmic antibody (ANCA)-associated vasculitis (AAV) due to methimazole use in a 64-year-old woman. She was initially hospitalized for abdominal pain and possible colitis, and subsequently developed hematuria, renal failure, and hemoptysis. The serologic work-up revealed positive antinuclear antibody (ANA) and perinuclear-antineutrophilic cytoplasm antibodies (p-ANCA), with positive antimyeloperoxidase. Three weeks following admission, the patient was found to be pulseless, and expired. At autopsy, microscopic review included widespread transmural necrotizing vasculitis and crescentic glomerulonephritis in the kidney, and diffuse pulmonary alveolar hemorrhage; focal coronary artery intimal vasculitis and necrotizing pericarditis were also noted. Several drugs have been associated with the development of ANCA-positive diseases, including propylthiouracil, hydralazine, allopurinol, penicillamine, and levamisole in cocaine. Association of ANCA vasculitis with methimazole exposure is less known, and severe presentation with fatal outcome, as seen in our patient, is exceedingly rare. We reviewed clinical and histopathologic features of drug-induced ANCA vasculitis associated with methimazole to raise awareness of this potentially life-threatening complication associated with this agent.

## Case presentation 

A 64-year-old female with a history of hypertension, asthma, pulmonary fibrosis, and hyperthyroidism secondary to multinodular goiter presented with abdominal pain and diarrhea and was admitted for possible colitis. On admission, she was noted to have acute kidney injury (AKI) with serum creatinine (Scr) of 2.8 mg/dL (baseline Scr was 1.2 mg/dL). She denied use of nonsteroidal anti-inflammatory drugs, proton pump inhibitors, or herbal medications; however, she reported having completed a course of clarithromycin for a respiratory infection 2 weeks prior. Her only home medication at the time of presentation was methimazole, which she had been taking for ~ 2.5 years (5 mg/p.o). She denied smoking or the use of illicit drugs, and her social history was otherwise nonrevealing. Subsequently, she developed gross hematuria with worsening AKI. 

Laboratory data revealed an elevated serum C-reactive protein, white blood cell (WBC) leukocytosis of 29.1 × 10^3^/µL, blood urea nitrogen was 50 mg/dL, Scr increased to 5.67 mg/dL, and potassium was elevated to 7.2 mg/dL. Serological work-up was positive for antinuclear antibody (ANA, 1:640), perinuclear antineutrophilic cytoplasmic antibodies (p-ANCA, 1:320), and myeloperoxidase antibody (MPO, 109.4), and negative for human immunodeficiency virus, hepatitis B, hepatitis C, rheumatoid factor, ribonucleoprotein antibody, double stranded (ds)-DNA antibody, Sjogren SSA and SSB antibodies, and antiglomerular basement membrane (GBM) antibody. Serum complement levels were within normal limits; C3 = 99 mg/dL (reference range 81 – 157) and C4 = 34 mg/dL (reference range 13 – 39). Serum free light chain ratio was not elevated, and serum immunofixation did not show any monoclonal gammopathy. Urinalysis showed 173 red blood cells, 13 WBCs, and spot urine protein to creatinine ratio was elevated at 1.2. Additionally, a chest X-ray showed bilateral pleural effusions. Kidney ultrasound revealed increased bilateral cortical echogenicity with bilateral hydronephrosis. The patient was started on empirical therapy for *Clostridium difficile* due to her diarrhea, but the test for *C. diff* toxin was negative. CT of abdomen was performed that was negative for infectious processes. She also underwent urine culture, stool culture, and blood cultures testing multiple times, however, they were all negative. Stool was also negative for ova, parasites, and protozoa. 

Given the negative anti-GBM and ds-DNA antibodies, normal complement levels, and positive ANCA serologic test with anti-MPO specificity in this patient presenting with pulmonary-renal syndrome, ANCA-driven vasculitis and pauci-immune crescentic glomerulonephritis was at the top of our differential diagnosis. Foley catheter was placed, and she was initiated on pulse dose corticosteroids due to clinical suspicion of ANCA)-associated vasculitis (AAV). She subsequently underwent kidney biopsy, which showed severe necrotizing small vessel vasculitis and crescentic glomerulonephritis, consistent with AAV. Methimazole was discontinued. Three days after the kidney biopsy, the patient developed hemoptysis and was initiated on plasmapheresis for concern of pulmonary alveolar hemorrhage. She eventually became oliguric, requiring hemodialysis. Few days later, she underwent change in mental status and eventually coded. cardiopulmonary resuscitation was unsuccessful, and the patient expired. 

## Pathology evaluation 

**Figure 1.**
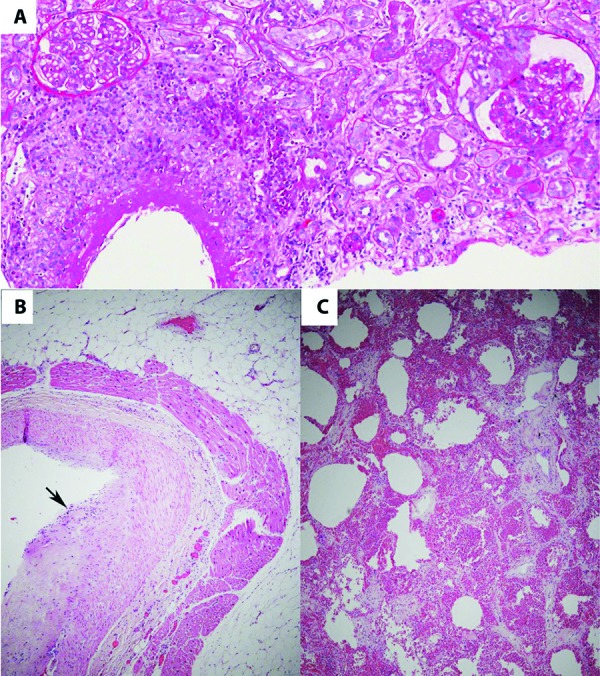
Histopathologic findings include: A: Necrotizing leukocytoclastic vasculitis with crescent formation in the glomerulus to the right, on a kidney biopsy performed days prior to the patient’s death (PAS stain, × 100). B: focal intimal coronary artery vasculitis (arrow; H & E-stained section, × 40). C: Diffuse pulmonary alveolar hemorrhage (H & E-stained section, × 40).

Following the patient’s death, an autopsy was performed. Microscopic evaluation of the kidney parenchyma was compatible with the findings of the recent prior kidney biopsy; there was widespread necrotizing leukocytoclastic vasculitis, with extensive transmural necrosis and polymorphonuclear cell infiltration (Figure 1). The light microscopy sample contained 30 glomeruli, 2 of which were globally sclerosed. Approximately 20% of the glomeruli revealed cellular crescents. Uninvolved glomeruli did not show significant changes; the capillaries were of normal thickness and texture, the mesangium was only segmentally prominent, and no significant hypercellularity was seen in endocapillary or mesangial spaces. Immunofluorescence studies performed on sections of paraffin-embedded tissue revealed nonspecific reactivity. No significant immune type glomerular deposits were seen on electron microscopy. Chronic changes were mild. 

Autopsy examination also revealed widespread pulmonary hemorrhage and focal subintimal vasculitis of a coronary artery (Figure 1). There was also focal necrotizing pericarditis adjacent to an involved artery. Other organs did not show significant changes that could explain the clinical course. 

## Discussion 

AAV is a group of small-vessel vasculitides, encompassing granulomatosis with polyangiitis (GPA), microscopic polyangiitis (MPA), and eosinophilic granulomatosis with polyangiitis (EGPA) [1]. There are two major ANCA autoantibodies – the cytoplasmic (c-ANCA), which confers antigen specificity for proteinase 3, and perinuclear (p-ANCA), with specificity for MPO; the cytoplasmic and perinuclear forms refer to the pattern of reactivity seen by indirect immunofluorescence test on alcohol-fixed test cells exposed to patients’ serum-carrying ANCA antibodies. ANCA-related vasculitides are often idiopathic, however, infections and drugs are the most common triggers for onset of this disease. 

In the retrospective analysis of ANCA-related vasculitis, patients with the highest anti-MPO antibody titers were reviewed for the use of commonly implicated offending drugs; 60% (18 of 30) of patients had been exposed for a minimum of 9 months (and in some cases for many years) to hydralazine, propylthiouracil (PTU), penicillamine, allopurinol, or sulfasalazine, frequently with renal involvement, and sometimes with biopsy-proven crescentic glomerulonephritis [2]. Apart from variability in dose and length of drug exposure, there is no good correlation of antibody titer with severity of presentation and the number of organs involved; in addition, the appearance of ANCA antibodies in serum and onset of ANCA-associated vasculitis are not necessarily correlated [3]. 

Methimazole is less commonly associated with the ANCA-related vasculitis than PTU. In a large study of ANCA-related complications associated with these 2 drugs, the adverse reactions were commonly characterized as severe, with 1 fatal outcome among 23 patients exposed to methimazole [3]. The lack of awareness of the association of methimazole use with ANCA-related disease may have resulted in lower recognition rates of the milder forms. The use of methimazole has been increasing with the growing prevalence of thyroid disease, raising the risk for methimazole complications. Other than vasculitis, commonly cited complications include arthritis, agranulocytosis, autoimmune hepatitis, cholestasis, hypoglycemia, pancreatitis, and hypoprothrombinemia [3]. The vasculitic process often involves kidneys and lungs [4, 5]. Other target organs include joints, muscles, nerves, or skin, with variable clinical presentation [3, 6]. 

The pathogenesis of methimazole-induced AAV is still not fully understood. The mechanism of PTU-induced ANCA disease has been studied with more interest, and over the years, several hypotheses have been entertained. Some of them include the notion that PTU accumulates in neutrophils, binds to MPO, and causes its alteration to a more antigenic form [7]. Another theory suggests that sera from patients that develop AAV in association with PTU have high reactivity towards specific MPO fragments when compared to those without AAV [8]. More recent research suggests that excessive activation of neutrophils by ANCAs induces formation of neutrophil extracellular traps (NETs), which are involved not only in ANCA-mediated vascular injury but also in the production of ANCAs themselves [9]. Nakazawa et al. [10] showed that PTU can induce abnormal conformation and degradation of the NETs, suggesting that PTU plays a direct role in AAV pathogenesis. A similar process may exist with methimazole exposure, although further studies are needed to confirm this mechanism of pathogenesis. 

Early recognition of this complication is critical, since discontinuation of the offending drug can not only lead to improvement in kidney function, but results in overall clinical recovery [11]. In patients where major organ injury is present, immunosuppression may be needed to suppress the inflammation and prevent permanent damage. Drug-associated ANCA vasculitis should be considered in patients with a history of culprit drug exposure, high-titer anti-MPO antibodies, and the presence of other autoantibodies including antinuclear, antihistone, antiproteinase 3, antielastase, antilactoferrin, or antiphospholipid antibody [2]. The presence of these antibodies is not a feature of idiopathic ANCA-associated disease [2]. ANCA-related glomerular disease is typically pauci-immune, however, it can be associated with immune complex deposits; the presence of an immune complex-mediated component on a kidney biopsy, in what otherwise appears as an ANCA-driven process, should prompt further investigation of drug exposures. Patients treated with methimazole should be monitored for serological evidence of ANCA antibodies and clinical manifestations of AAV, regardless of the dose and length of drug exposure. Patient survival and the risk of end-stage kidney disease are closely associated with degree of renal dysfunction at time of presentation. Physicians, including endocrinologists, pulmonologists, and nephrologists, should be aware of this potentially life-threatening, severe adverse event related to methimazole use. 

## Funding 

No funding was provided for this manuscript. 

## Conflict of interest 

The authors have no multiplicity of interests to disclose.
